# Demonstrating impact of allied health professional participation in the NIHR Associate Principal Investigator scheme

**DOI:** 10.1186/s12913-025-12584-1

**Published:** 2025-05-14

**Authors:** Florence Cook, Nicky Gilbody, Jenny Hunt, Zoe Knight, Heulwen Sheldrick, Lisa Houghton, Caroline Ewers, Michael Caygill, Holly Speight, Irwin Nazareth, Roganie Govender

**Affiliations:** 1 Head & Neck Centre, University College London Hospital NHS Foundation Trust, 250 Euston Road, London, NW1 2PG UK; 2 Head and Neck Academic Centre, Division of Surgery and Interventional Science, University College London, Charles Bell House, 43-45 Foley Street, London, W1W 7TS UK; 3 North Middlesex University Hospital NHS Trust, Sterling Way, London, N18 1QX UK; 4 Luton and Dunstable Hospital, Bedfordshire Hospitals NHS Foundation Trust, Lewsey Road, Luton, LU4 0DZ UK; 5 The Clatterbridge Cancer Centre, Clatterbridge Road, Bebington, Wirral CH63 4JY UK; 6 Liverpool University Hospitals NHS Foundation Trust, Aintree Hospital, Lower Lane, Liverpool, Merseyside L9 7AL UK; 7 South Tyneside and Sunderland NHS Foundation Trust, Sunderland Royal Hospital, Kayll Road, Sunderland, SR4 7TP UK; 8 National Institute for Health and Care Research Associate Principal Investigator Scheme, 21 Queen Street, Leeds, LS1 2TW UK; 9 Department Primary Care & Population Health, University College London, Royal Free Site, Rowland Hill Street, London, NW3 2PF UK

**Keywords:** Clinical trial, Associate PI, Allied health professionals, Capacity building, Impact assessment model, Clinical trial delivery, Researcher development, Research impact

## Abstract

**Introduction:**

Research impact is defined as an effect, change or benefit to the wider society or services beyond academia. Measuring impact demonstrates benefit and value for money of publicly-funded research. This study evaluates differing levels of impact associated with completion of the National Institute for Health and Care Research Associate Principal Investigator (PI) scheme on SIP SMART2 (Swallowing Intervention Package - Self Monitoring, Assessment & Rehabilitation Training 2); cluster-randomised multi-centre phase II trial with a focus on Prehabilitation of swallowing in head and neck cancer.

**Methods:**

Data was acquired using two qualitative methods: Reflective virtual discussion group and documentary evidence based on the individual portfolios/checklists of eight accredited Associate PIs. Framework analysis and the evidence of impact model was employed for analysis.

**Results:**

High level impact was identified on the micro level, with evidence of individual learning and sense of pride in becoming an accredited Associate PI. Medium to high level impact was found at the meso level including taking a leading role in research delivery within own organisations and raising professional profiles amongst the wider team. There were limited examples directly demonstrating macro level impact.

**Conclusion:**

The Associate PI scheme provides opportunities for professional groups that otherwise might not be involved in clinical trials, promoting equality and inclusiveness with benefits across multiple levels of impact. The current checklist of activities is designed to demonstrate competence in clinical trial delivery and may not currently capture the wider benefits and impact of the scheme. These could be better captured with some additions to the checklist including follow-up on potential impacts accrued beyond the 6-month timefame.

**Supplementary Information:**

The online version contains supplementary material available at 10.1186/s12913-025-12584-1.

## Introduction

**Fig. 1:**
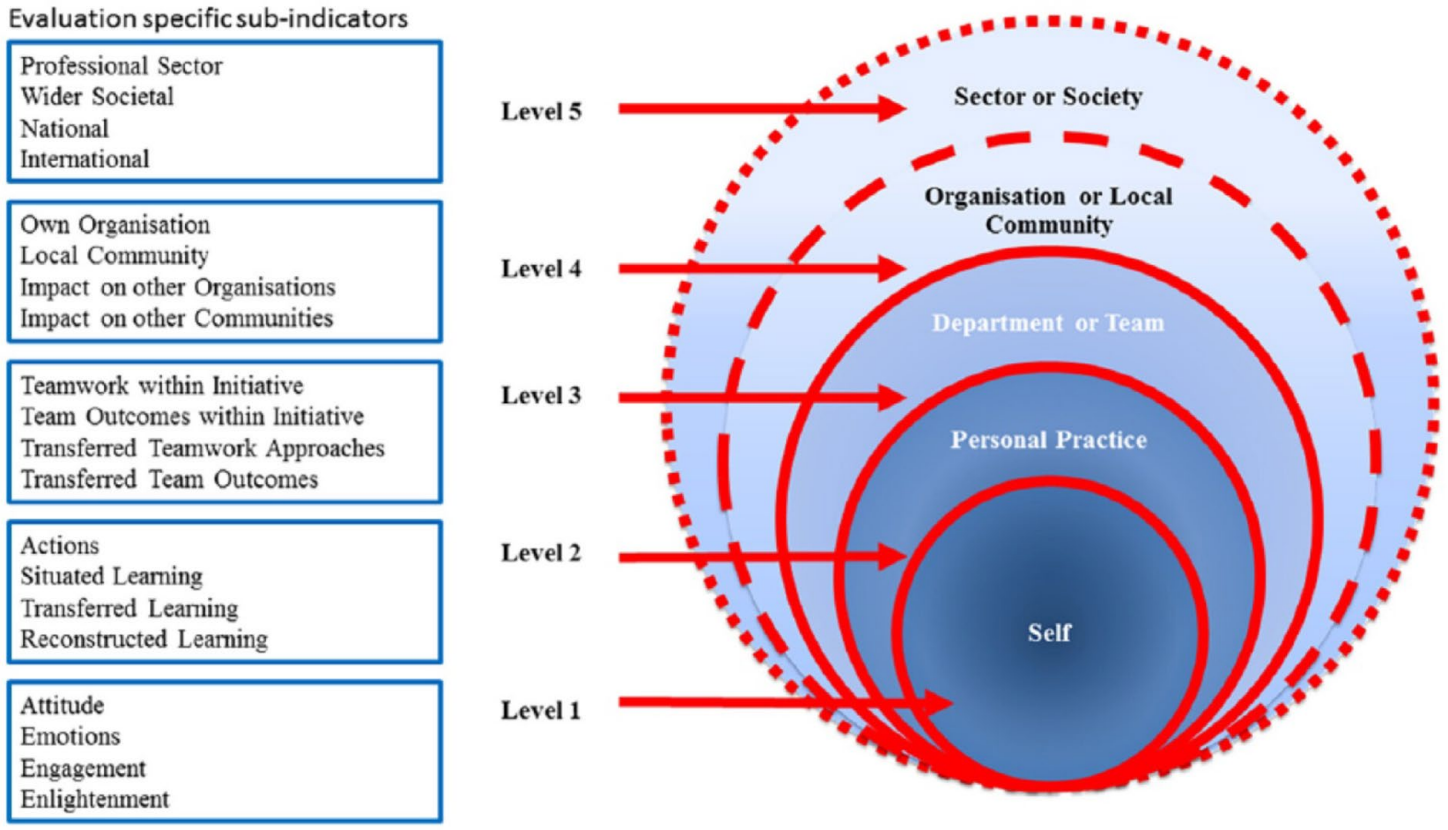
The evidence of impact model (reproduced under license CCBY4.0). Source: [7]

Research impact has many definitions [1]. The United Kingdom Research Excellence Framework defines impact as *‘an effect on*,* change or benefit to the economy*,* society*,* culture*,* public policy or services*,* health*,* the environment or quality of life*,* beyond academia’* [2]. Measuring impact is increasingly expected of researchers to demonstrate benefit and value for money, especially when research is funded from the public domain. Different approaches exist to measuring research impact and the choice depends on the type of research conducted and philosophical underpinning. For example, the Payback Framework [3] consists of a logic model of the research process from conceptualisation to impact and a series of five categories that classify payback including knowledge, benefits to future research, health and the health system and economic benefits. The Research Impact Framework [4] on the other hand, was designed to capture different types of impact (including research, policy, practice, service and societal) for use by individual researchers. Similarities between these frameworks include capturing impact at different levels, including impact on individual, organisational and societal level, alternatively referred to as ‘micro, meso and macro’. The Vitae Research Development Framework [5] was developed to support individual researchers track their development through capturing the behaviours, attributes and knowledge of successful researchers together with the skills and personal qualities that enable successful working with others to ensure the wider impact of research. The framework is structured into four domains which comprise (1) Knowledge and intellectual abilities (2) Personal effectiveness (3) Research governance and organisation (4) Engagement, influence and impact [6]. The evidence of impact model [7] is based on the existing concept of micro, meso and macro levels of impacts [8, 9] and was developed to capture the reach of impact from team-based capacity building within healthcare. The model (Fig. 1) [7] includes five distinct levels: impact on self and personal practice (levels 1–2; micro), impact on department or team, organisation or local community (level 3–4; meso) and impact on professional sector or wider society (level 5; macro).

In June 2023, NHS (National Health Service) England published the long-term workforce plan for the NHS in the UK [10]. This strategy included the importance of fostering skills to enable staff to increase research activity to support a culture of evidence-based practice. Ambitions have been set out to improve capacity and capability by empowering staff to lead, participate and deliver research thereby increasing the number of clinical academics in the workforce to bridge the current gap between the NHS and academic partners [10, 11]. An example of this includes the National Institute for Health and Care Research (NIHR) Associate PI scheme; launched in November 2021, following a successful pilot. This scheme offers a 6-month in-work training opportunity that supports health and care professionals in developing research skills and delivering clinical trials under the mentorship of a local principal investigator [12].

As a relatively new scheme, there are limited studies to date which explore the impact associated with the Associate PI scheme. Cook and colleagues (2022) demonstrated how activities attributed to the scheme supported individual researcher development across all domains of the Vitae Research Development Framework [13]. Most recently, Newman and colleagues (2024) explored experiences and impact of the programme on trainee professional development alongside impact on trial recruitment. It was found that trial sites with Associate PIs recruited significantly more patients than sites without Associate PIs and that participating in the Associate PI scheme provided an educational opportunity to develop research skills [14]. Similarly, Jepson and colleagues (2021) explored experiences of the Associate PI scheme amongst surgical trainees and respective supervising PIs. Benefits derived from registering onto the scheme included encouraging a culture of research, development of research skills and supporting career development of trainees [15].

Limited literature has evaluated the wider impact of the scheme; and particularly amongst healthcare professionals outside of medicine such as nursing and allied health professionals (AHP). We therefore aimed to evaluate the impact of the NIHR Associate PI scheme using the Evidence of Impact Model in the context of SIP SMART 2 (Swallowing Intervention Package - Self Monitoring, Assessment & Rehabilitation Training 2) trial. SIP SMART 2 is a multi-centre phase II trial with a focus on Prehabilitation in swallowing for people with head and neck cancer (HNC) and was one of the first AHP-led trials registered on the NIHR Associate PI scheme.

Prehabilitation of swallowing refers to interventions that target swallowing muscles from the time of cancer diagnosis to commencing acute treatment, and may include physical and psychological components to reduce the severity of current and future impairments [16]. SIP SMART2 is a swallowing prehabilitation intervention package that comprises multiple components including educational counselling, tailored information, personalised exercises and strategies informed by behaviour change theory to support engagement with advice and exercise [17].

## Methods

The group undertook a virtual focus group discussion to facilitate reflective learning and practice [18] as part of SIP SMART2; a cluster-randomised multi-centre phase II trial (ISRCTN12377415) [19]. Ethical approval for SIP SMART2 was granted by the London Bridge Research Ethics Committee and registered with the Clinical Trial Registry (http://www.isrctn.org/) on 4th March 2022 (Clinical Trial Number: ISRCTN12377415). No additional ethical approval for this sub-study was required, as this was not deemed to be research but an evaluation of Associate PI experiences of undertaking the scheme. However, all Associate PIs had previously consented to participation in interviews that were part of the trial process evaluation. All Associate PIs (also co-authors) consented to the virtual discussions and agreed for their completed checklists to be included in this analysis. We followed the consolidated criteria for reporting qualitative research (COREQ) guidance [20] where appropriate for this study.

The NIHR Associate PI scheme is an in-work training opportunity to support junior clinicians gain experience in clinical trial delivery under the mentorship of a local PI. The scheme is open to any health and care professional in the United Kingdom, who is able and willing to commit to supporting delivery of a study at local level for a minimum six-month period. During this time, a checklist of mandatory study activities is required and on completion, formal accreditation of Associate PI status is issued from the NIHR, in recognition of engagement in NIHR portfolio research studies endorsed by the NIHR and Royal Colleges. The most up to date version of the checklist is publicly accessible and can be found online via the NIHR Associate PI toolkit website (https://drive.google.com/file/d/1XujVzMWB1BYqiP-019A0WJmIJ0QDWg0I/view). An example of a well completed checklist is also available publicly on the NIHR website (https://drive.google.com/file/d/1rTWVFFnr1LNaFP2mBMhwdoei9UWq_hf-/view). PIs can register studies onto the NIHR Associate PI scheme dashboard, where Associate PIs can then apply via the NIHR learn website and are selected/approved by the local PI [12].

### Sample

The chief investigator registered SIP SMART2 on the NIHR Associate PI scheme in April 2022. All sites were invited to participate in the scheme provided that the site PI was willing to provide mentorship. Five of the six study sites took up the offer (with 2 trusts having 2 Associate PIs who registered consecutively) between April 2022 and January 2023. Eight registered Associate PIs went on to receive formal accreditation with the first accredited in October 2022 and last October 2023. At the end of the patient recruitment window, all accredited Associate PIs were invited to participate in this current sub-study.

### Data collection

#### Individual portfolio checklists

Associate PIs were required to complete a checklist of mandatory study activities overseen and signed off by the site PI and a member of the national study team. Activities include essential skills such as recruitment, management of the investigator site file and undertaking Good Clinical Practice training. All Associate PIs (*n* = 8) agreed to participation by submitting their individual portfolio checklists for documentary analysis. Further to this, they also reported any relevant activities since completing the scheme allowing for the capture of impacts that may have occurred in the period shortly after accreditation as an Associate PI.

#### Virtual focus group discussion

The chief investigator and group facilitator (RG), an experienced clinician and qualitative researcher invited the first wave of accredited Associate PIs (*n* = 4) to form a “discussion group” to facilitate their ongoing learning via email. The group undertook a one-hour virtual reflective discussion audio-visually recorded via Microsoft Teams (Microsoft Office 365) to share their individual experiences of the Associate PI scheme and the benefits and challenges encountered. A structured topic guide was not utilised and an open-ended question was asked by the chief investigator at the start of the discussion, inviting group members to share their experiences of taking on the Associate PI role. As part of the iterative discussions and learning, members of the group agreed for discussions to be included for analysis and all reviewed the transcript.

### Analysis

A structured evaluation was conducted of the individual portfolios kept by the Associate PIs as part of their accreditation requirements for the NIHR Associate PI scheme together with the transcripts from virtual focus group discussions. Data from checklists were analysed by 2 Associate PIs separately (HS and LH) and from transcripts (JH and ZK) between January and February 2024. This involved extracting evidence of activities from the checklists and quotes from recordings against the domains within the evidence of impact model [7]. A simple matrix was developed drawing upon the Framework Analysis method [21] and data was then imported onto a single matrix. Multiple sources of data added robustness and triangulation. A pragmatic approach was used for interpretation as follows: the level of evidence pertaining to each level of impact was categorised as ‘high’ (frequent mention and inference in over 50% of checklists), ‘medium’ (occasional mention and inference featured in 25–50% of checklists) and ‘low’ (minimal mention and inference featured in less than 25% of checklists).

## Results

Table 1 delineates the demographics of the eight Associate PIs. All were from an allied health background with the majority speech and language therapists (87.5%) and one dietitian (12.5%). The majority were female (87.5%) and levels of post-registration experience varied from 5 to 10 years to 30–40 years.

**Table 1:** Demographics of Associate PIs

**Characteristic**	**Details of Associate PIs n (%)**
**Age**	
18 – 24	0 (0)
25–34	2 (25)
35–44	1 (12.5)
45–54	3 (37.5)
55–64	2 (25)
**Gender**	
Male	1 (12.5)
Female	7 (87.5)
**Ethnicity**	
White or White British	7 (87.5)
Asian or Asian British	0 (0)
Black, Black British, Caribbean or African	0 (0)
Mixed or multiple ethnic groups	1 (12.5)
Other	0 (0)
**Clinical background**	
Speech and Language Therapist	7 (87.5)
Dietitian	1 (12.5)
**Post-registration experience (years)**	
0 - 5	0 (0)
5 – 10	1 (12.5)
10 – 20	3 (37.5)
20 – 30	2 (25)
30 – 40	2 (25)
**Education level**	
Undergraduate degree	3 (37.5)
Postgraduate degree	5 (62.5)
Masters	4 (80)
PhD	1 (20)

Data for *n*=8 Associate PIs

Table 2 demonstrates a summary of how levels of impact within the evidence of impact model were met from the completed Associate PI checklists. The full matrix of examples is available in Supplementary file 1.

### Micro level impact

Generally, a high level of evidence (frequent mention and inference in over 50% of checklists) was demonstrated for individual level impact which was consistent with the virtual focus group recordings. It was outside the scope of the checklist to provide opportunities for disclosure of participant perspectives on being an Associate PI however, it was evident from the virtual focus group discussion that individuals experienced pride and a sense of achievement from participation:

“huge huge learning and I guess, the bit about for me, yes, it did give me personal satisfaction and it has challenged me beyond belief and I’ll take that forward into other things as well” [Participant 1].

“Our research and development lead, I think she’s been quite surprised by how competent we can be as AHPs and in fact, she commented that my site folder had been put together better than quite a lot of other people who actually do research as a day job…I think the positives that have come out of this are showcasing our skills, which doesn’t happen often and it’s brilliant” [Participant 2].

Medium levels of evidence (occasional mention and inference featured in 25–50% of checklists) indicated situated, transferred and reconstructed learning (see level 2 on evidence of impact model for definition) which was supplemented with evidence from the virtual focus group discussion. This included the learning involved of both trial set up, and governance around measuring patient-reported outcome measures:

“Then that learning curve around really understanding what’s involved in setting up research locally, Like you were saying (Participant 3), the amount of paperwork involved. I had no idea” [Participant 1].

“I think the other key piece of learning on a personal level was what outcome measures are because I hadn’t sat down with a patient and taken consent from them for a trial and run through questionnaires with them… It’s made me more cautious of how I run through questionnaires with patients and how I am conscious of about not influencing something they might say” [Participant 3].

### Meso level impact

A medium level of evidence (occasional mention and inference featured in 25–50% of checklists) was demonstrated for department/team and organisational level impact which was consistent with the virtual focus group recordings. This included evidence of team working within own organisations. Virtual focus group discussions indicated the impact this had in terms of raising profile within the wider multi-disciplinary team for taking a leading role in research and being asked to be Associate PIs and/or co-PIs on other studies:

“We’ve got quite a high profile in our MDT (multidisciplinary team) and its been a way of maintaining that profile for other members of the team and encouraging those discussions within the MDT that actually someone else is taking a lead on research and raising that profile amongst the consultant staff” [Participant 4].

“I am already being asked if I would consider being an API (Associate PI) for another research study” [Participant 1].

“Once my competence and confidence in this (Associate PI role) had been reviewed they said, ok you can step up to the PI role” [Participant 2].

### Macro level impact

Sector or macro level impact showed a low level of evidence (minimal mention and inference featured in less than 25% of checklists) which again was consistent with the virtual focus group recordings. Examples of impact on the checklists included dissemination of the scheme locally and nationally. The role of being an Associate PI contributing to research that drives evidence-based practice was discussed in the virtual focus group, highlighting a further macro level impact:

[On creating more efficiencies in delivery of the clinical trial] “I think that is a skill of AHPs, isn’t it? What we do well as an AHP… I think that’s underappreciated, particularly within research and development… we do have lots of skills that can equally contribute to research” [Participant 1].

“my manager for example… I think this is the first time he’s heard of the API scheme and I think the fact I have been an API and I’ve gone down this process… it’s raised awareness of the level of involvement we can have on research as an AHP…if someone else came to him and asked him about the API scheme, he would know more about it because he knows that I’ve done it and what I’ve learned” [Participant 3].

**Table 2:** Evidence of impact model applied to Associate PI checklists

Level of impact		Checklist evidence
1: Self	Micro	Implied evidence of being motivated and committed but little evidence about how participants felt about being an Associate PI. High evidence of engagement e.g. organisational skills, networking across the wider team and working with the research team on a regular basis.
2: Personal Practice	Micro	High evidence of enlightenment e.g. knowledge acquisition of research governance. Medium evidence of situated, transferred and reconstructed learning e.g. response to change as a result of increase knowledge e.g. deviation to protocol. High evidence of action-based learning e.g. taking leadership in managing the research process.
3: Department or Team	Meso	High evidence of teamwork and working across different clinical teams and members of the research team.
4: Organisation or Local Community	Meso	Medium evidence of impact in own organisation e.g. upskilling staff. No evidence of impact on other organisations or communities.
5: Sector or society	Macro	Low evidence of disseminating findings to professional sector. No evidence of wider societal or sector impact.

Table 3 depicts activities of Associate PIs up to one year following completion of the scheme. This indicates individual/micro level impact through using the scheme to support professional development as an advanced clinical practitioner role and application for a doctoral fellowship award. Organisational/meso level impact has been reported through taking on PI roles locally. On a societal/macro level, the scheme has been disseminated on a regional and national level, through professional groups and a journal publication. This has impact in terms of increasing awareness of clinical research opportunities available whilst in practice and the skills that may be acquired from undertaking the Associate PI scheme.

**Table 3:** Examples of impact following completion of the Associate PI scheme

Level of impact		Examples from activities following completion of the scheme
1: Self	Micro	Professional development: • Associate PI accreditation used as evidence against research pillar for an advanced clinical practice e-portfolio. • Associate PI accreditation as an output/research achievement for a NIHR Doctoral Fellowship Application (which was subsequently awarded). • Skills acquisition from Associate PI scheme used to support (1) writing a research grant application (2) completion of an application for funding to present abroad.
2: Personal Practice	Micro	
3: Department or Team	Meso	Leadership in research within trusts: • Stepping up as PI and/or taking on co-PI role for SIP SMART2. • Taking on PI role on other studies. • Increased working with Research and Development teams locally.
4: Organisation or Local Community	Meso	
5: Sector or society	Macro	Dissemination: • Local: presenting at local educational event in cancer • Regional: Sharing experience ofbeing an Associate PI and supervising CI/PI at a NIHR clinical research network webinar professional group meeting. • National: Sharing experience of the Associate PI scheme at: (1) national NIHR conference aimed at people new to research to increase awareness of clinical research activities whilst in practice (2) national head and neck oncology conference aimed at multidisciplinary staff working in head and neck cancer. • Journal publication comparing skills acquisition from undertaking the Associate PI scheme with a well-known research development framework (vitae RDF).

*Abbreviations*: *Vitae RDF* Vitae Research Development Framework, *PI* Principal Investigator

## Discussion

This study aimed to evaluate the impact of the NIHR Associate PI scheme using the evidence of impact model in the context of the SIP SMART 2 trial. Findings demonstrate that there is significant impact associated with participation in the Associate PI scheme at the micro level, with evidence to support individual acquisition of research skills and confidence. Impact was also demonstrated at a meso level, with benefits to organisations including teamwork and upskilling staff in research delivery. Impact appeared more limited at the macro level but did include local and national dissemination of the scheme and journal publications demonstrating the research skills that can be acquired from the scheme.

To date, involvement in research has been more commonly associated with medical professions, with nursing, midwifery and allied health professionals reporting increased difficulty commencing a clinical academic career path [22, 23]. Our findings demonstrate the significant impact of the Associate PI scheme on an individual level. The scheme aids continued professional development (CPD) through establishing and facilitating an entry point or foundation on which to build or extend research experience. Career progression was positively influenced, with Associate PI qualification was seen to enhance funding applications, leading to successful attainment of further training awards to undertake doctoral study and used as part of a portfolio of work evidencing advanced level practice. Moreover, findings revealed that participation increased the likelihood of AHP adoption of further PI or co-PI roles. Previous research with medical trainees demonstrated the advantages of the Associate PI programme for early research career development [14, 15]. Our study has similarly found positive benefits for AHPs.

We also found evidence of upstream benefits where positive impacts at an individual level may yield associated impacts at meso level. For example, we noted the sense of pride and satisfaction reported by individuals performing Associate PI roles within organisations. Job satisfaction has been linked with improved performance in healthcare staff [24] which, in turn, has been associated with improved patient outcomes [25]. Involvement in the Associate PI scheme may also result in workforce benefits. Indeed, education and development opportunities, both professional and academic, have been associated with improved recruitment and retention in nursing staff [26] and AHPs [27]. Given the current NHS-wide workforce crisis with reduced staffing levels across medical and allied health professions [28], inclusion of such development opportunities in job descriptions may present an attractive recruitment incentive and reduce staff turnover.

Furthermore, our meso-level findings revealed use of the scheme to establish AHP inclusion in organisational research roles, both within individual Trusts and across wider research networks. Providing AHPs with opportunity and training in research skills may widen the pool of researchers available across networks. This could facilitate participation in clinical trials and enhance trial delivery by improving recruitment. Indeed, improvements in trial recruitment and efficacy have previously been linked with participation in the Associate PI scheme [7].

Evaluation of macro level impact is more difficult to quantify at such an early stage, however wide dissemination of information through conference presentations, educational events and publication may form the foundations for increasing numbers of AHPs in research in future, leading to increased research participation across healthcare networks. Involvement of healthcare systems in research has been associated with improved patient experience, improved quality and efficiency of care and reduced mortality [29], thus a societal impact may be seen in future. Further, registering the scheme across multiple centres ensures availability of opportunity across geographical locations, thus improving the diversity of the research network which is key to addressing inequalities in clinical research [30]. Capturing impact at the macro level is imperative for standardising the scope of roles within regulatory frameworks and securing the funding and support necessary to enable more AHPs to participate in research initiatives such as the Associate PI scheme [31]. Impact may be demonstrated through narrative case studies involving stakeholder feedback, describing the journey from research to impact and effects [32], reporting longitudinal outcomes for at least five years after the event [33], sharing of ‘big data’, such as supplementing Researchfish entries (the online reporting system for research outcomes and impact by Elsevier) with bibliometrics [1], and establishing advocates for research in leadership positions where they may be able to influence attitudes and behaviours around instilling a culture of research within clinical settings [34].

### Limitations

The individual portfolio design of the Associate PI checklist aims to demonstrate competence in clinical trial delivery, and is not necessarily modelled to demonstrate broader impact. Information from checklists may therefore miss the wider impact of completing the Associate PI scheme that go beyond an individual/micro level. This work is also limited by a single group discussion at one time point; however, we hope that by demonstrating activities post-completion we have captured impact beyond immediate completion. Furthermore, findings are limited to a small sample of Associate PIs mainly from speech and language therapy backgrounds due to the specifics of the SIP SMART2 clinical trial. However, given the multicentre nature of the trial we believe our findings are likely generalisable to centres across the UK. Finally, examples of impact post participation in the scheme were only captured for one year, and it is plausible that further macro level impact has yet to be realised. System change within large organisations, such as the NHS, is a notoriously lengthy process [35], with common delays between the emergence of peer-reviewed publications demonstrating the value of a particular approach and resultant uptake into policy and practice [36]. Additional supportive research may take years to appear, and individual candidates require time and opportunity to act upon information disseminated to date. As such, follow up at additional future time points is recommended, to capture this translation of knowledge into practice.

### Future work

This study provides new information which can be used to guide future research. More in-depth exploration of experiences is required, with the aim of learning how the scheme can be optimally adopted across healthcare systems. This includes studies capturing the long-term impact of participation and whether involvement as an Associate PI leads to ongoing engagement in research. The NIHR are also exploring ways to track Associate PI career progression following scheme completion. Future direction of the scheme has included establishing additional alumni learning resources to support ongoing development and individuals are also offered further support through the Workforce Leads in each Regional Research Delivery Network. Further opportunities may include those that consolidate skills as an Associate PI and/or further develop as a PI. This may be supported by local programmes or national schemes such as the NIHR Principal Investigator Pipeline Programme. This is currently limited to research nurses and midwives but is being encouraged to open to other professional groups [37].

Our study has also highlighted that greater consideration should be given to the design of the scheme as an in-work training activity. Reliance on support from an existing PI may exclude those in under-resourced teams with little or no allocated CPD time and those who have limited access to clinical trials. At the same time, participation in such a scheme may help garner commitment to establish and recognise research as part of a professional role. Further thought on how to identify sites that might benefit most and how to enable and incentivise uptake is warranted.

Wider research into the applicability of the scheme to healthcare services with more limited access to existing PIs for support is warranted, with exploration of potential frameworks to support inclusion. Further, investigation into whether participation in the scheme results in formal allocation of research time in job roles may demonstrate further meso-level impacts. Additionally, analysis of AHP research opportunities across acute, outpatient and community settings may reveal inequalities, where introduction of this scheme and collaboration between services may yield benefits across healthcare systems.

On a wider level, exploration of whether increased numbers of AHPs in research enhances trial recruitment and delivery and whether this is associated with an impact on patient outcomes may support a hypothesis of potential macro-level impacts of the scheme.

## Conclusion

This study has illustrated the benefits of participation in the Associate PI scheme and demonstrated how this can have positive impact at an individual, Trust and network level. We have shown that AHPs are well placed to build research capacity and deliver on trial implementation, with added personal benefits in terms of career development and job satisfaction. We hypothesise that wider societal impacts may be seen in the future, including increased numbers of AHPs in research with consequent improvement in patient outcomes.

To date, AHPs have been a largely untapped source of potential in research, with a wealth of skills relevant to the effective delivery of clinical trials. This study has highlighted that greater AHP involvement yields a wide range of benefits and, as such, should be prioritised among research teams.

## Supplementary Information

Supplementary Material 1

## Data Availability

The datasets used and/or analysed during the current study are available from the corresponding author on reasonable request.

## Abbreviations

AHP: Allied Health Professional
Associate PI: Associate Principal Investigator
PI: Principal Investigator
HNC: Head and neck cancer
CPD: Continued professional development
NIHR: National Institute for Health and Care Research
SIP SMART: Swallowing Intervention Package - Self Monitoring, Assessment & Rehabilitation Training
