# Supplementary material for: Demonstrating impact of allied health professional participation in the NIHR Associate Principal Investigator scheme
Source: BMC Health Serv Res. 2025 May 14;25:700. doi: 10.1186/s12913-025-12584-1 (PMC12080059; doi:10.1186/s12913-025-12584-1)
Supplement: Supplementary file 1 — Supplementary Material 1 [file 12913_2025_12584_MOESM1_ESM.docx]

Results of impact from NIHR Associate PI checklists and recordings on the SIP SMART 2 trial from Evidence of Impact Model

| **Impact Domain** | **Sub indicator** | **Theme (checklist)** | **Evidence (checklist)** | **Description** | **Quotes from checklist** | **Quotes from recordings** |
| --- | --- | --- | --- | --- | --- | --- |
| **Self** | **Attitude** | **Motivation** | Medium | Implied evidence of being motivated and committed (through actions & activity) – but not explicitly stated | ‘I gave positive feedback to the team around how we can be actively involved in national clinical trials within our practice...’ | 1. ‘the personal challenge and that first decision; should I? shouldn’t I? can I, can’t I?’  2. ‘when we submitted the feasibility, I didn't expect actually for you to come back and say yes please. I think I was expecting not to probably hear back at all’ |
| **Self** | **Emotions** | **Feelings** | Low | Very little report of how participants FELT about being involved in the API process. Some expression of perseverance – but implied rather than stated | - None | 1. ‘being reassured that I don’t have to answer questions that they ask me just because I can’  2. ‘so huge huge learning and …it did give me some personal satisfaction and it has challenged me beyond all belief’  3. ‘there's the skills I think that it made me look at what I think I can do well. Perhaps also the things I need to develop as well’  4. ‘I found it a really frustrating process and really slow because we'd had a bit of chopping and changing in our department’  5. ‘manage the research within our day-to-day practice and I think that's been a challenge, but also quite rewarding. And I am proud of the team for doing it as well. I have to say’  6. ‘A huge challenge for me was not having done anything like this before’  7. ‘Feeling proud of myself as I’m the first person in our hospital accredited as an API’ |
| **Self** | **Engagement** | **Practical issues** | High | Organisational skills. Many descriptions of contacting people, updating records etc | - ‘Meeting with’ - ‘Liaising with’ - ‘Supporting….’ | 1. ‘It's been that proud to speak up a bit more as well’  2. ‘once my competence and confidence in this had been reviewed they said, ok you can step up to the PI role because of the trial itself’ |
| **Self** | **Engagement** | **Networking** | High | Clear and regular evidence of networking within and across teams – SLT, R&D team, CNS, radiography, Consultant staff. Evidence of working with APIs, PIs and CI on regular basis | - ‘Meeting with’ - ‘Liaising with’ - ‘Supporting….’ |  |
| **Self** | **Enlightenment** | **Developing skills and knowledge** | Medium | Evidence of learning and changing processes as a result of increased knowledge – e.g. deviation to protocol, managing SIV etc | - ‘Feeding back to the team….’ - ‘Amending the processes…’ | 1. ‘makes you think a lot about what QOL is and what types of questions they ask you as a result of the questions you are asking them’  2. ‘research nurse…how she approaches questions people ask her has been quite informative for me and being reassured that I don’t have to answer questions that they ask me just because I can’  3. ‘the amount of paperwork involved. I had no idea’  4. ‘influencing the questionnaires … keep it very much on them and stop the therapist within me who wants to say this/that/the other and start to fix things and just accept their responses as they are’  5. ‘big learning curve, I am also doing an MRes at the moment and the difference between the paperwork and the set up, the processes for a small piece of research that I am doing compared to a clinical trial is mind blowing’  6. ‘reflecting on the challenges on time management’ |
| **Personal Practice** | **Actions** | **Logistics/**  **planning** | High | Much documentation of series of steps, processes and roles – who does what and when | - ‘Meeting with’ - ‘Liaising with’ - ‘Supporting...’ | None |
| **Personal Practice** | **Actions** | **Leadership role** | High | Lots of evidence of the API taking the lead in various activities to communicate, monitor, promote, document and manage the research process | - ‘Leading the SLT team in….’ - ‘Presenting the study at...’ | None |
| **Personal Practice** | **Situated Learning** | **Knowledge** | High | Clear acquisition of knowledge about research pathway, process, design and management of protocol deviations | - ‘Documenting…’ - ‘Recording...supporting the SLT team’ | 1. ‘my personal learning, there was lots that I didn’t know about trials. I had never come across a delegation log, a training log, a screening log’.  2. ‘learning curve around really understanding what's involved in setting up research locally and I think...the amount of paperwork involved’.  3. ‘consenting someone for a research trial feels very formal in that way, in a way that sometimes taking consent in other ways doesn't feel quite as formal’.  4. ‘influencing the questionnaires and that being a huge learning curve just in that sometimes they want your input ‘aww should I said this or this?’ |
| **Personal Practice** | **Situated Learning and transferred learning** | **Problem solving** | Medium | Dealing with deviations to protocol or instances when patients wished to withdraw from the study. Evidence of increased awareness of general research process | - ‘Through discussion with the CI...’ - ‘The CI widened the criteria for eligibility for the screening log. This was communicated to the research team. It demonstrated that changes can be made as the study evolves and are sometimes necessary to meet the study objectives’ | 1. ‘made me more cautious of how I run through questionnaires’  2. ‘so the informed consent was really useful. Taking ideas from that and understanding the process of seeking consent from a patient. And … that skill will go with me and probably with my team, not just when you're involved in research, but when we are consenting patients and involved in the process of consent even for some of their big surgeries and treatment that they're having’.  3. ‘I don't think I'll ever not remember that you need to initial on a consent form’. |
| **Personal Practice** | **Reconstructed learning** | **Problem solving** | Medium | Dealing with deviations to protocol or instances when patients wished to withdraw from the study. Evidence of increased awareness of general research process | - ‘Through discussion with the CI...’ - ‘The CI widened the criteria for eligibility for the screening log. This was communicated to the research team. It demonstrated that changes can be made as the study evolves and are sometimes necessary to meet the study objectives’ | 1. ‘I reflected a little bit on the skills that I have and the things I think I'm reasonably good at, which is managing bigger projects’ |
| **Departmental or team** | **Teamwork within initiative** | **Working as a team** | Medium | Clear and regular evidence of teamwork within and across teams – SLT, R&D team, CNS, radiography, Consultant staff. Evidence of working with APIs, PIs and CI on regular basis | - ‘Meeting with’ - ‘Liaising with’ - ‘Supporting…’ - ‘Emailing…’ | 1. ‘already going to impact on the team because it isn't just about me if we participate in this research’  2. ‘being the go-to person for the members of the team when they're coming to ask questions about it’  3. ‘so how can we do that as a team?... What processes do we have in place? How can we fit this in? How can we make things more efficient without compromising the trial’  4. ‘so that's actually made us work really efficiently within that and keeps us very water tight within the time frames of consenting patients’.  5. ‘so again, I think it pulled us together even more … as a team, used our skills within the team because …it's recognising your team members and what certain people are very good at’. |
| **Departmental or team** | **Teamwork outcomes within initiative** | **Problem solving** | Medium | Dealing with deviations to protocol or instances when patients wished to withdraw from the study. Evidence of increased awareness of general research process | - ‘Through discussion with the CI…’ - ‘The CI widened the criteria for eligibility for the screening log. This was communicated to the research team. It demonstrated that changes can be made as the study evolves and are sometimes necessary to meet the study objectives’ |  |
| **Departmental or team** | **Transferred teamwork approaches** | **Grant application** | Low | One member of the team only – applying for a PPI research grant | - None | 1. ‘I'm already being asked if I would consider being an API for another research that's happening within probably ENT’.  2. ‘raising profile amongst other members of the team and what she was saying about being asked about being an API on other studies’.  3. ‘it it's been a way of maintaining that profile for other members of the team and encouraging those discussions within the MDT that actually someone else is taking a lead on research and raising that profile amongst the consultant staff’.  4. ‘they sort of fed back to me some of the things that they've learned as a result of doing GCP training and more about the ethics about research’.  5. ‘my line manager…if someone else came to him and asked him about the API scheme, he would know more about it because he knows that I've done it and what I've learned’. |
| **Departmental or team** | **Transferred team outcomes** | **Research dissemination** | Low | One member of the team only – leading on and presenting at H&N conference | - None | 1. ‘in subsequent meetings with our research and development lead, I think she's been quite surprised by how competent we can be as AHPs and in fact, you know, she commented that my site folder had been put together better than quite a lot of other people who are actually do research as a day job’. |
| **Organisation or local community** | **Own organisation** | **Upskilling team in research** | Medium | Evidence that acquisition of knowledge about research pathway, process, design and management of protocol deviations was shared with the SLT and R&D team | - None | 1. ‘I think the positives that have come out of this are that showcasing of our skills … but also now that we have got those links, we've got those links with the research team’.  2. ‘I think you were actually asked not to be another API but a Co-PI rather than an API on another big study… of surgically driven study…and you are all now absolutely leaders in this and the people you are working with are recognising it’.  3. ‘Our consultant is so very supportive and keep saying really you are doing the job of a PI…that has been appreciated’. |
| **Organisation or local community** | **Local community** |  | None |  | - None | None |
| **Organisation or local community** | **Impact on other organisations** |  | None |  | - None | None |
| **Organisation or local community** | **Impact on other communities** |  | None |  | - None | None |
| **Sector or society** | **Professional sector** | **Disseminating findings** | Low | One member of the team only – leading on and presenting at H&N conference | - None | 1. ‘I guess raised awareness of the level of involvement we can have and research as an AHP’.  2. ‘at the moment the API scheme is something that we do alongside our clinical roles…whether or not that will change at all in terms of funding time for clinicians to be able to be involved in research because it can be quite challenging to juggle’.  3. ‘in the future we could look at a model where clinicians do get more time built into their roles to facilitate research’.  4. ‘equally contribute to research’  5. ‘I think it is more recognising these skills that we’ve learnt that are recognised in advanced practice for things like prescribing another other areas in terms of research delivery’.  6. ‘The advanced clinical practitioner e-portfolio program… provide evidence that you are performing at an advanced level for each of the pillars…As AHP’s we could all be going down advanced clinical practitioner routes and a scheme like the API scheme or involvement in clinic trials is going to really support that status’. |
| **Sector or society** | **Wider societal** |  | None |  | - None | 1. ‘seeing people's reactions on Twitter, for example, I have a lot of positive feedback on Twitter … by doing the scheme’. |
| **Sector or society** | **National** |  | None |  | - None | 1. ‘equally contribute to research’  2. ‘I would take this forward … to the CRN because AHP trials are not as well supported as the big pathology trials, for example’.  3. ‘This undervaluing us has really come through’ |
| **Sector or society** | **International** |  | None |  | - None | None |

^Abbreviations: AHP = Allied Health Professional; API = Associate Principal Investigator; CI = Chief Investigator; CNS = Clinical Nurse Specialist; CRN = Clinical Research Network; ENT = Ear Nose Throat; GCP = Good Clinical Practice; H&N = Head and Neck; MDT = Multidisciplinary team; PPI = Patient and Public Involvement; SIV = Site Initiation Visit; SLT = Speech and Language Therapist.^
